# SaVeBRAIN.Kids—study protocol for a cluster-randomized stepped-wedge trial to reduce hospitalizations for mild traumatic brain injury in children in Germany

**DOI:** 10.1186/s13063-025-09240-8

**Published:** 2025-10-30

**Authors:** Nora Bruns, Pia Brensing, Linda von der Heiden, Christian Dohna-Schwake, Simone Schwarz, Johanna Wagner, Eva-Maria Huessler, Michael Nonnemacher, Anja Neumann, Frederik Valbert, Silke Neusser, Roman Peter, Dirk Reinel, Joerg Scheidt, Yannic Siebenhaar, Johannes Drescher, Florian Wogenstein, Laura Hoerster, Berit Schoppen, Ala Edin Abdin, Sven Marx, Petra May, Anika Hüsing, Andreas Stang, Florian Heinen, Michaela Bonfert

**Affiliations:** 1 Department of Pediatrics I, Neonatology, Pediatric Intensive Care Medicine, Pediatric Neurology, and Pediatric Infectious Diseases, University Hospital Essen, University of Duisburg-Essen, Essen, Germany; 2 Centre for Translational Neuro-and Behavioural Sciences, C-TNBS, University Hospital Essen, University of Duisburg-Essen, Essen, Germany; 3 Department of Pediatric Neurology and Developmental Medicine and LMU Center for Development and Children with Medical Complexity, Dr. Von Hauner Children’s Hospital, University Hospital, LMU Munich, Munich, Germany; 4 Centre of Clinical Epidemiology, Institute of Medical Informatics, Biometry, and Epidemiology, University Hospital Essen, Essen, Germany; 5 Institute for Health Care Management and Research, University of Duisburg-Essen, Duisburg, Germany; 6 Institute for Health Care Management and Research in Essen (EsFoMed), Essen, Germany; 7 Institute of Information Systems, University of Applied Sciences Hof, Hof, Germany; 8 Smartlytic GmbH, Hof, Germany; 9 MedEcon Ruhr GmbH, Bochum, Germany; 10 Smx Consulting, Pfullingen, Germany

**Keywords:** Pediatric, Mild traumatic brain injury, Hospitalization, Novel care pathway

## Abstract

**Background:**

Traumatic brain injury (TBI) is one of the most important pediatric conditions worldwide. In Germany, hospitalization rates for mild TBI drastically exceed hospitalization rates from similar healthcare systems.

**Methods:**

The SaVeBRAIN.Kids trial will implement and test a novel care pathway (nCP) for evidence-based standardized risk assessment, structured observation in the emergency department (ED) for several hours, and technology-supported home monitoring with the aim to reduce hospitalizations. This non-inferiority multicenter study will be carried out using a cluster-randomized stepped-wedge design, with all centers starting in the control phase and sequentially transitioning to the intervention. Eligible participants (age ≥ 3 months and < 18 years) must present within 48 h of head injury, have minimal symptoms (Glasgow coma scale ≥ 14), and no risk factors for intracranial complications. The co-primary outcomes are the relative risk of hospitalization and the proportion of unplanned re-visits within 72 h of presentation to the ED for ambulatory cases. Secondary outcomes include clinical safety measures, cost-effectiveness, and process evaluation. Based on power calculations (*α* = 0.05, power = 0.9), 1390 patients will be recruited over 12 months.

**Discussion:**

The SaVeBRAIN.Kids trial addresses a relevant healthcare challenge by testing a new approach to pediatric mild TBI management in Germany. It aligns with current evidence while accounting for the country’s specific healthcare context. If successful, the intervention could substantially reduce unnecessary hospitalizations and free inpatient capacities while preserving patient safety.

**Trial registration:**

German Clinical Trials Registry (DRKS00035623). Registered on January 21, 2025.

**Supplementary Information:**

The online version contains supplementary material available at 10.1186/s13063-025-09240-8.

## Introduction

Traumatic brain injury (TBI) is defined as the presence of neurological symptoms indicating brain dysfunction and/or injury to the brain, skull bones, or adjacent structures resulting from mechanical force on the head [1]. Symptoms range from mild headaches to deep unconsciousness and can occur isolated or as complex symptom patterns. Based on the presence/extent of initial state of consciousness, TBI is classified into three severity grades: mild, moderate, or severe, with mild TBI (mTBI) significantly dominating pediatric cases at 97%.

In Germany and worldwide, TBI represents one of the most epidemiologically and health-economically relevant pediatric conditions, accounting for approximately 92,000 pediatric hospital admissions per year in Germany. Of these, approximately 2500 (2.7%) are diagnosed with imaging-confirmed intracranial injury/complications, and only about 600 (0.7%) require neurosurgical intervention [2]. Notably, Germany’s pediatric TBI hospitalization rate of 687/100,000 child-years (CY) (1457/100,000 CY for infants) is the highest reported in literature worldwide [2]. In contrast, the UK reports 280/100,000 CY [3], and the USA merely 73/100,000 CY [4].

The high hospitalization rates in Germany are likely attributable to insufficiently standardized local care pathways for mild TBI, resulting in clinical uncertainty during decision-making among healthcare providers. The current German national guideline for “TBI in Childhood and Adolescence” broadly recommends inpatient monitoring, particularly for children under 2 years of age. Vague recommendations regarding difficult-to-objectify symptoms like mild or transient consciousness impairment, headaches, nausea, or dizziness add up with lacking options to monitor the short-term clinical course in the emergency department.

As a result, inpatient observation is often carried out despite lacking medical necessity. Further, the median duration hospital stay of 2 days for children with mild TBI exceeds the guideline-specified timeframe for intracranial complication manifestation (6–12 h). Contrasting the German practice, the USA guideline recommends no monitoring for patients at low risk for intracranial complications, while guidelines in the UK, Scandinavia, and the Netherlands recommend only hourly monitoring in a clinical setting for these patients [5–8].

The SaVeBRAIN.Kids trial will evaluate a novel care pathway (nCP) for children with mTBI that was designed to (1) standardize management of mild TBI; (2) implement the option of a short-term symptom-oriented observation in the emergency department (ED) instead of immediate hospital admission; (3) support caregivers during home monitoring via a specifically designed smart phone app. The aim of the nCP is to reduce the proportion of hospitalizations among pediatric patients with mTBI, who are at very low risk for relevant intracranial complications while maintaining equivalent medical safety to the current standard of care. The study will be carried out as a non-inferiority trial using a multi-centered cluster-randomized stepped-wedge design with the hospitalization rate and unplanned re-visits within 72 h from ED discharge as co-primary outcomes.

## Methods

### Participants, interventions, and outcomes

#### Study setting

**Fig. 1:**
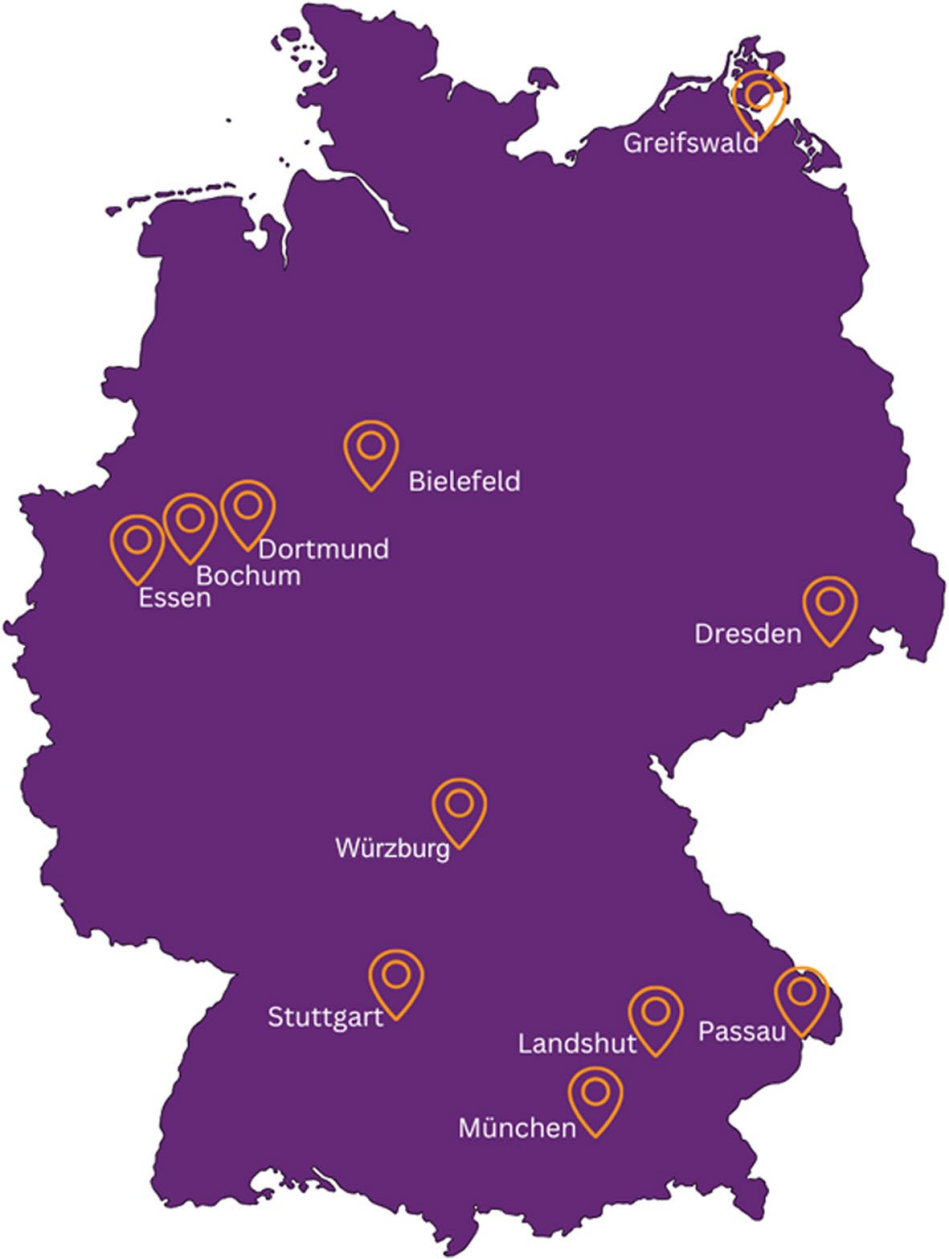
SaVeBRAIN.Kids study sites

Eleven pediatric study sites in Germany will participate in the study, including university hospitals, non-university tertiary care centers, and community hospitals from across the country (Fig. 1). Eleven study sites were recruited on a first-come-first-serve basis. Individuals performing the intervention at the study sites will be local investigators that oversee the local ED staff (physicians and nurses).

#### Eligibility criteria

Admission to the nCP requires a strict pre-selection of patients with a very low risk of clinically relevant intracranial complications. The risk assessment for a clinically relevant intracranial injury is based on the Glasgow Coma Scale (GCS) and further evidence-based clinical risk factors that are suitable to identify children with intracranial injuries [9]. The clinical factors chosen for SaVeBRAIN.Kids are based on the symptoms defined in the PECARN rule (Pediatric Emergency Care Applied Research Network (PECARN) Head Injury Decision Rule) and the German guideline [1, 10], as well as the clinical course during the monitoring period.

Key eligibility criteria are age ≥ 3 months and < 18 years, head trauma or mild TBI within ≤ 48 h of presentation to the ED, and the absence of risk factors (Table 1).

**Table 1:** Inclusion and exclusion criteria, mandatory monitoring, and inpatient admission requirements for pediatric patients with mild traumatic brain injury (TBI) in the emergency department

**Inclusion criteria**	• Age ≥ 3 months and < 18 years, who have an ICD-10 code of S06.0, S06.8, S06.9, or S02.0 • Head injury caused by direct or indirect mechanical force • At least one neurological symptom related to the head injury was/is present and/or there is a simple skull vault fracture • GCS ≥ 14 upon arrival in the emergency department • The structured medical history and examination reveal no increased risk or indication of a clinically relevant intracranial complication • The trauma occurred ≤ 48 h ago • The legal guardians have sufficient German language skills for providing the medical history, understanding information about the mild traumatic brain injury, and using the parent app • A suitable device is available for the legal guardians to use the parent app
**Exclusion criteria**	• Unconsciousness > 30 min • Head injury in the context of high-impact trauma • Head injury due to an explosion (so-called blast injury) • Penetrating injury • Open traumatic brain injury (TBI) • Known bleeding tendency/indication of a coagulation disorder • Known severe internal, oncological, or hematological underlying condition associated with an increased risk of complications or bleeding • Known (developmental) neurological underlying condition • Difficulty in reliably assessing the neurological status of the patient • Presence of a shunt/valve in cases of known elevated intracranial pressure/hydrocephalus • Use of medications that impair blood coagulation • Indications of intoxication (alcohol/medication/drugs/other substances) • Indications of injury with suicidal intent • Indications of child abuse • Indication for hospital admission due to associated injuries or social/structural reasons • Occupational accident insurance case and private health insurance case, as these are excluded by the sponsor statutory health insurance members with the last digits 4 and 9
**Monitoring in ED mandatory**	• Initial high symptom burden in the anamnesis • Brief loss of consciousness (≤ 30 min) • Impaired consciousness, disorientation, or amnesia • Any neurological abnormalities in the detailed neurological examination ◦ Cervical spine: Spontaneous pain in the cervical spine/neck area, pain with movement, restriction of movement range, or pain upon palpation ◦ Cranial nerve abnormalities ◦ Abnormal spontaneous motor activity ◦ Coordination abnormalities in following test: Finger-to-nose test or age-appropriate grasping, arm holding test, diadochokinesis, finger opposition test, gait pattern, toe/heel walking, Romberg stance, one-leg stance, tightrope walking ◦ Muscle weakness in upper or lower extremities ◦ Abnormal muscle tone ◦ Pathological muscle reflexes (BSR, PSR, ASR)
**Inpatient admission mandatory**	• Parents feel unsure about going home • The parents’ app does not work on the smartphone • High burden of symptoms with no improvement or worsening during monitoring in the ED • Newly emerged pathological findings during monitoring in the ED • Medical concerns from the physician regarding discharge to home • Trauma-related relevant pathological findings in imaging

#### Interventions

The trial is carried out using a cluster-randomized stepped wedge design. The 11 study sites will participate in the study over 12 months. All study sites will start in the control phase and one study site (cluster) per month will switch to the intervention arm, which will be carried out until the end of the study. The switching order will be randomized, and study centers will be informed 8 weeks in advance to switching.

##### Control group

During the control phase, the standard care pathway (sCP) is carried out according to each study site’s current clinical practice, including inpatient or outpatient management. Local standard operating procedures will be collected prior to the beginning of the study. Screening and clinical documentation will be performed using the study’s electronic case report form (eCRF). After 72 h “unplanned re-visits” will be queried via telephone follow-up by the local study team and documented in the electronic case report form (eCRF).

##### Intervention group

**Fig. 2:**
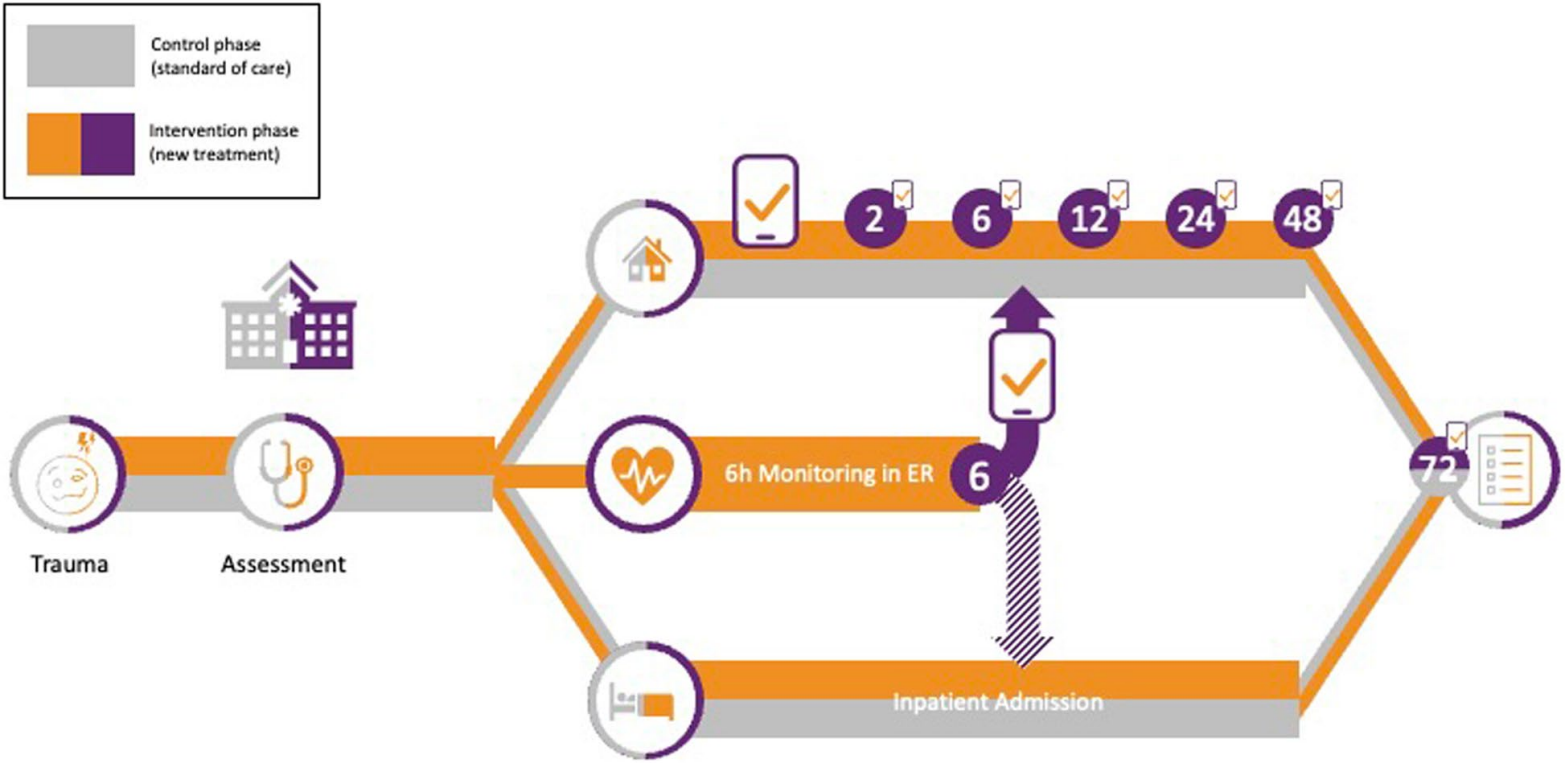
Participant timeline. Inpatient admission and primary outpatient management are available throughout the study and constitute the standard care pathway. After 72 h, follow-up to assess unplanned re-visits and parents’ satisfaction with the treatment is carried out. During the intervention phase, an additional option of a 6-h observation period in the emergency department is added for patients that cannot immediately be discharged from the ED due to their clinical condition. At the end of the observation period, the final decision is made for either inpatient admission or secondary outpatient management. All parents (regardless of inpatient or primary or secondary outpatient management) receive a parent app on their smartphones. In outpatients, the parent app will send push notification to remind parents to assess their child’s condition at pre-specified timepoints (2, 6, 12, 24, and 48 h after presentation to the ED). All parents will receive a push notification to fill out the 72-h follow-up. If the follow-up is not answered via the parent app, parents will receive a phone call by the local study team as in the control phase

The intervention phase is designed to test the nCP, which provides the additional option of secondary outpatient management for low-risk patients after a symptom-oriented observation period in the ED (Fig. 2 and supplementary figure). Upon inclusion during the intervention phase, one of the patients’ caregivers receives a smartphone app designed to facilitate monitoring their child in the home environment.

##### Observation in the ED

The nCP consists of symptom-based clinical neuromonitoring for at least 6 h starting at ED arrival and includes the assessment of the Glasgow Coma Scale (GCS), pupillomotor function, and vital signs. The frequency and duration of the monitoring and additional measures depend on the patient’s health condition upon arrival at the ED and during monitoring (Table 1). Hospital admission, imaging, or surgery are possible at any time as indicated according to clinical findings and clinical practice guidelines. Patients that remain stable during the nCP will be discharged home if the physician and the legal guardians agree that the child is fully conscious (GCS = 15), behaves age-appropriately, and does not display newly developed symptoms or demonstrates any focal neurological deficits. Pre-existing symptoms, such as headaches or vomiting, must have remained stable (without worsening), show signs of improvement, or have resolved during the monitoring period. The legal guardians must feel confident in conducting home monitoring, and the parent app must function on their smartphone. Additionally, adequate medical care in case of emergency must be ensured through a functioning emergency response chain or by the guardians’ ability to arrive at the ED. Deviations from the recommended care pathway are possible at any time but will be documented along with the reason (Table 2).

**Table 2:** Reasons for deviations from the study protocol, including logistical, technical, medical, and personal factors

**Deviations from study protocol**	• Logistical reasons • Technical reasons • Admission at parents’ request • Discharge against medical advice • Study termination by the parents • Medical concerns from the physician • Others (string)

##### Monitoring in the home environment

Caregivers receive a push notification at pre-specified times (2, 6, 12, 24, 48 h after discharge), requesting them to assess their child’s health status. Included items are general condition (alertness, behavior for children up to 3 years old, or orientation for children 3 years and older), pupillomotor function, motoric function, eating/drinking behavior for children up to 3 years old or headaches for children 3 years and older, and nausea/vomiting. The assessment is age-adapted and uses binary answers that are structured so that critical responses can be easily identified. Caregivers are instructed to contact the treating ED if they have answered any item with a critical response. The app will not interact with parent’s responses and does not have a built-in alarm function. All recommendations by the app will be displayed statically already before responses have been entered. Each inquiry will remain open to answer until the next scheduled inquiry.

#### Definition of outcomes

The primary outcome consists of two co-primary outcomes (CPO) to assess the effectiveness of the intervention in reducing hospital admissions and the safety of the nCP in terms of unplanned re-visits. Secondary outcomes comprise clinical safety outcomes, healthcare economic evaluation, and aspects of health service delivery.

##### Primary outcomes

 1st Co-primary outcome: Relative risk of hospitalization among all included cases (outpatient and inpatient) at initial ED presentation.

2nd Co-primary outcome: Proportion of cases with unplanned re-visit within 72 h after discharge from the ED.

##### Secondary outcomes

Secondary hospitalization following initial outpatient management, re-visits (all-cause and TBI-related), missed clinically relevant intracranial injury, admission to intensive care unit, neurosurgical intervention, death, costs/cost-effectiveness, acceptance, and barriers to implementation.

#### Sample size calculation

The sample size calculation considered both CPOs, using generalized linear mixed models (GLMM) for both analyses. To estimate the relative risk of hospitalization, a GLMM with log link is planned. The calculation is based on hospitalization data from 2022 (ICD-10 German modification S02.- and S06.-) from 14 study sites that provided ED data on ambulatory care and hospitalizations during the pre-planning of the study with a mean admission rate of 61% (range [22%, 93%]). For the sample size calculation, a mean hospitalization rate of 50% was assumed to account for possible changes in hospitalization behavior due to implementation training or fluctuating admission capacities. The study’s aim is a relative risk reduction of 20% (relative risk = 0.8). The GLMM also requires the intra-cluster correlation coefficient (ICC), which is estimated at 0.142 based on available data (Fleiss-Cuzick estimator) and set at 0.15 for the calculation. With a power of 0.9, *α* = 0.05, and 11 study sites a sample size of 1056 patients is required for the first CPO.

The second CPO is examined using a non-inferiority test. A GLMM with identity link will be applied to estimate the risk difference. Based on an unplanned re-visit rate of 6% in one of the participating study sites and literature-reported rates of 1.5% [11], an average unplanned re-visit rate of 3% is assumed before the intervention. A 2-percentage point increase in unplanned re-visits during the intervention is anticipated due to potentially increased parental need for reassurance. In addition, a 5-percentage point increase in unplanned re-visits is considered medically acceptable, as patients would otherwise still be in hospital, and spot-ED presentations are considered less burdensome than hospitalization. Thus, an unplanned re-visit rate in the nCP that ≤ 7 percentage points above standard care is considered non-inferior. With an assumed ICC of 0.1, power of 0.9, *α* = 0.025, and 11 participating study sites, a sample size of 1320 (10 per study site per month) is needed. Due to the very short observation period, a drop-out rate of 5% is assumed, resulting in a required sample size of 1390 (11 per study site per month).

The leading sample size is derived from the non-inferiority testing plus the assumed dropout rate is established as the recruitment target for the prospective clinical study.

#### Recruitment

The participating study sites collectively attend 8.326 cases per year (reference year, 2022) with an average of 757 annual and 63 monthly cases. According to the sample size calculation, 11 patients per study site per month should be enrolled, representing approximately 35% of the eligible case volume after subtracting 50% of the total case number due to assumed ineligibility. To account for regional and seasonal variations of TBI incidence and recruitment success, a maximum number of 13 recruited patients per center per month will be allowed.

The study’s core team has ensured close engagement of the study sites throughout the grant application process and will continue to do so by providing continuous on-site and virtual support, information material, and financial compensation for recruitment. If recruitment difficulties arise in one study site, higher case numbers could be permitted for one or more well-recruiting study sites, or additional study sites could be onboarded.

Caregivers of potentially eligible patients will receive study information immediately during the initial triage by the ED staff, thereby making use of the waiting time for preliminary information.

### Assignment of interventions and blinding

The randomization of the clusters will be carried out by one of the independent study’s evaluators (Institute for Medical Informatics, Biometry, and Epidemiology of the University Hospital Essen; IMIBE) using appropriate software. The core team will receive information on the start date with appropriate lead time and pass this information on to the study sites that will enroll patients.

The core team and attending local ED staff cannot be blinded for the assignment group, because implementing and performing the nCP involves their active participation. All evaluators will be blinded for the assignment group until the end of analyses. Unblinding will be performed by a trusted third party (TTP).

### Data collection, management, and analysis

#### Data collection and management

Clinical data including the two co-primary outcomes (CPO) are documented via the eCRF that includes a trial participant management system for the attending physicians (hospitalization, re-visit at the same hospital) or via a final query in the parent app/telephone follow-up (re-presentation at different hospital): If a patient returns to the same hospital within 72 h after discharge home, the patient case is reactivated in the eCRF and relevant treatment data is recorded, including time of presentation; reason for presentation; presentation related to head injury; consequences (imaging, outpatient management, hospitalization, type of hospitalization, need for neuro/cranial surgery); and outcome (neurological status at discharge, death). For phone calls from caregivers within 72 h after initial presentation, the case is reopened in the eCRF and documentation is supplemented with information about call time, reason, relation to head injury, and consequences by the attending physician.

During home monitoring in the intervention group, parents will enter data times at the time points specified to assess their child’s condition (2, 6, 12, 24, and 48 h after ED presentation) for the study documentation.

In routine clinical care, patients may present to hospitals other than their initial treating facility. For this purpose, an automated query is sent via the parent app 72 h after presentation to the ED. This final query requests information about whether caregivers have visited an ED, hospital, emergency services, or pediatrician within 72 h after discharge from the ED. If confirmed, follow-up questions address whether the visit was related to the head injury and its consequences. If the query is not answered within 5 to 7 days after discharge in the smartphone app, parents will receive a phone call to verify the endpoint “unplanned re-visit” by the local study team. The control group receives their phone call 72 h to 7 days after presentation to the ED with the documentation as for the intervention group.

For cases with fatal outcomes after the 72-h active query period, passive surveillance remains possible through reporting by participating sites until the completion of data collection for the entire study.

After meeting the monthly recruitment target, the local study physicians will document eligible but non-approached cases using the same software as for the study documentation anonymously.

Additionally, anonymized retrospective data collection will be performed as a historical control cohort at each study site, covering a 12-month period before initiation of the SaVeBRAIN.Kids project (September 2023–August 2024). This control cohort serves as a comparison group to assess possible effects attributable to project training and use of the structured eCRF. The inclusion criteria for this cohort are the same as for the control- and intervention group. This data is transmitted to the TTP by each study site to verify that no personal data is included, concatenated, and forwarded to the clinical evaluator (IMIBE) for statistical analyses.

The basis for the healthcare economic evaluation is billing data from the enrolled patients who are insured with one of the consortium’s statutory health insurances. This includes the pseudonymized merging of the routine data with clinical data, which were documented during the survey period in the eCRF and parent app, at the TTP. The routine data for outpatient physcian visits, medical aids, medication, rehabilitation measures funded by statutory health insurance, and hospital stays are included. The expenses for the nCP compared to standard care will be estimated through personnel deployment, using time stamps in the eCRF. Additionally, investment, material, and personnel costs for the implementation of the intervention, as well as the costs for staff training, will be assessed. The overall cost analysis from billing data is conducted for the period 1 week before and after the trauma, with sensitivity analysis 1 month before and after the trauma.

**Fig. 3:**
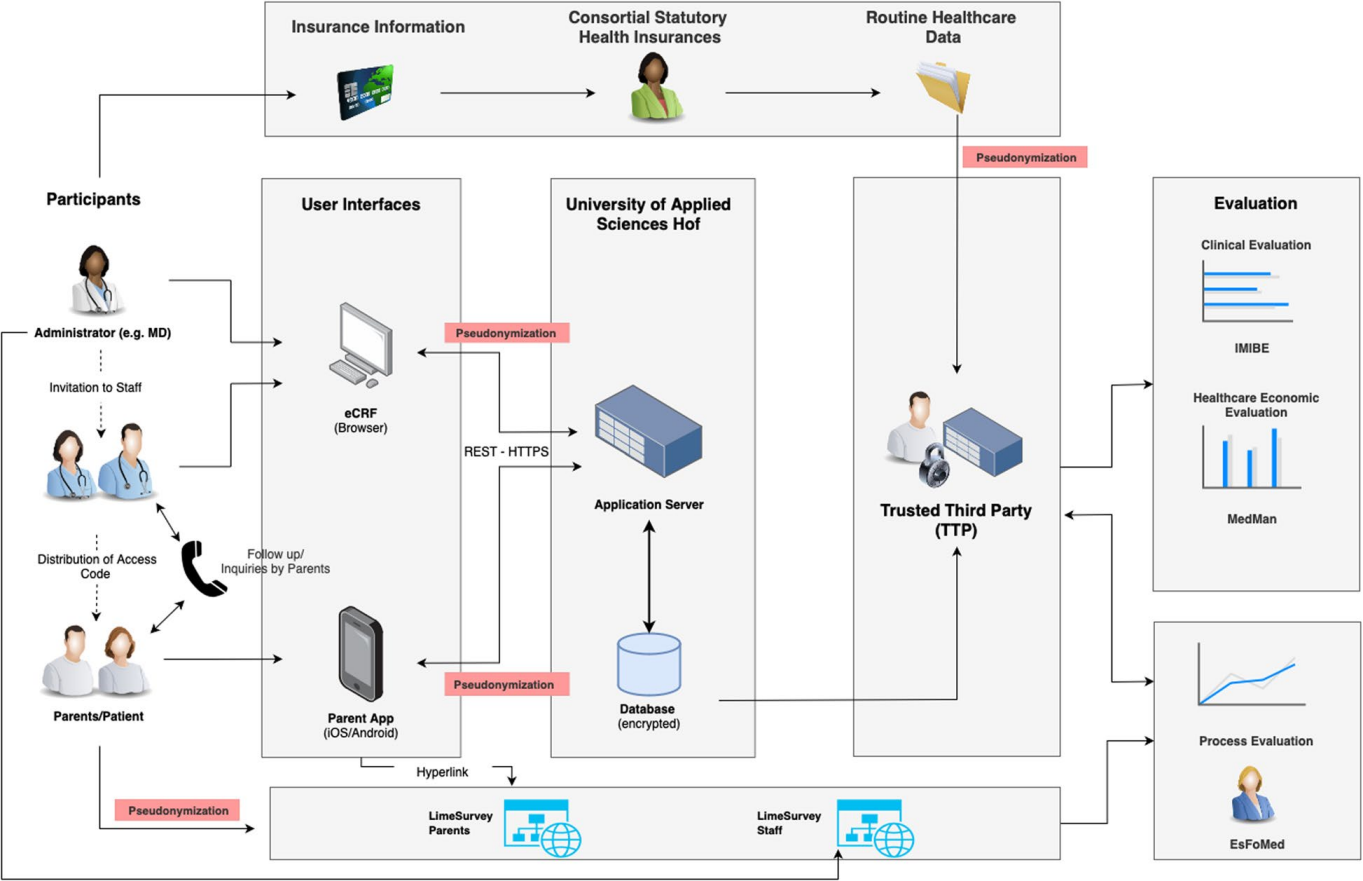
Data flow. Clinical data are entered into the eCRF by the local investigators at the study site and by the parents into the SaVeBRAIN.Kids parent app. The eCRF for the physician interface contains mainly single- or multiple-choice options and includes plausibility ranges and cross validation for continuous data. Pseudonymized clinical data from the eCRF and parent app are stored on a server at Hof University and forwarded to the TTP. The study sites transmit patient data and corresponding pseudonyms to the patients’ respective health insurances, who in return transmit billing data to the TTP for merging with clinical data based on the pseudonyms. After resolving plausibility issues, the final datasets will be forwarded to the clinical and economic evaluators for their planned analyses. The parent survey for process evaluation will collect pseudonymized answers via an online survey hosted by EsFoMed. To enable descriptive and adjusted analyses, the TTP will forward basic demographic information to EsFoMed. Staff surveys will be collected via an anonymous online survey hosted by EsFoMed. For all survey data, plausibility checks will be performed by EsFoMed

During treatment and observation in the ED, the following time stamps are automatically recorded in the eCRF, which will be used for the health economic evaluation: beginning and end of (a) medical history taking, (b) of the body check, (c) of the neurological examination, (d) until the further procedure is initiated, and (e) of symptom-oriented monitoring (Fig. 2). Additionally, in each ED time spent on collecting GCS, pupil reflexes, and vital signs as part of the monitoring process will be assessed for 10 patients to derive the costs associated with the monitoring process. As part of the process evaluation, parents will be surveyed regarding their acceptance of the medical measures and potential improvements via an eCRF in LimeSurvey (LimeSurvey GmbH, Hamburg, Germany) using their child’s study pseudonym. Letters requesting participation will be handed out by the study sites, containing a QR code for accessing the online survey and for the parent app users a directed link after the 72-h questioning will appear. ED staff will be queried via anonymous LimeSurveys. The data flow throughout the project is detailed in Fig. 3.

Monthly audits will ensure that recruitment targets are met, and appropriate individualized measures will be undertaken to enhance recruitment if necessary. Regular plausibility checks of entered clinical data including audit trails will be applied to ensure data quality. For health insurance data, plausibility and quality checks will be conducted at the TTP and issues resolved in bilateral correspondence with the respective health insurance company. For survey data, plausibility checks will be performed by the Institute for Health Care Management and Research. Additional data cleaning will be performed as necessary before analyses by the respective evaluators if not done by the TTP a priori.

#### Statistical methods

##### Clinical evaluation

For the hospitalized patient group, the relative risk change due to the nCP for the binary endpoint “hospitalization” will be estimated using a GLMM with log link and modeling fixed effects for intervention and time as well as random study site effects. A Wald Z-test will be used to test the null hypothesis of equal hospitalization risk with and without the intervention at a significance level of *α* = 0.05. For the non-inferiority analysis with respect to the proportion of patients with unplanned re-visits, the absolute risk difference will be considered. A GLMM with an identity link for the binary endpoint of unplanned re-visits, again with fixed effects for intervention and time and random study site effects, will be applied. A one-sided 97.5% confidence interval will be estimated for the difference in unplanned re-hospitalization rates. If the non-inferiority margin is not overlapped by the confidence interval, the null hypothesis of inferiority will be rejected. The two endpoints will be tested co-primary, i.e., both statistical tests have to be significant for a study success. As co-primary testing does not inflate the type I error rate, there is no need to correct for multiple testing, while an overall power of more than 80% can be preserved assuming that the two tests are independent each with a power of 90% (power of 1 st COP times power of 2nd COP).

Secondary outcomes will be analyzed using appropriate statistical methods as specified in the statistical analysis plan (SAP) prior to the beginning of the study. Clinical documentation will be performed in the software tools designed for the SaVeBRAIN.Kids study to ensure high data quality.

Primary analyses will be conducted as intention to treat, comparing outcomes in patients recruited before and after the study sites’ staff introduction to the nCP according to the randomization. Study sites with major protocol violations (e.g., late or no training for the nCP) will be excluded from a sensitivity analysis of a per-protocol study population. Further details will be specified in the SAP.

Due to the very short observation period for the primary outcomes, a drop-out rate of less than 5% is assumed. The primary analyses will thus be conducted as complete-case analysis. Sensitivity analyses will be conducted in which missing primary outcomes will be replaced with either a success (best-case scenario) or failure (worst-case scenario). The reason for missing values in the primary outcomes will also be recorded. In the unexpected case that more than 5% of values are missing, it will be possible to differentiate whether the missing data is likely to be Missing at Random (MAR) or Missing Not at Random (MNAR). For observations with more than 5% values missing under the MAR assumption, multiple imputation methods may be applied, and the results compared with the complete-case analysis. Further handling of missing data will be defined in the SAP.

To detect changes that may result from increased awareness merely due to study participation, anonymous retrospective data collection is carried out in terms of a historical control cohort, covering a 12-month period before the beginning of the project at each study site. In this case, the outcome parameter is the relative number of hospitalizations out of the total number of cases that would potentially meet the study’s inclusion criteria. This measure does not prevent potential bias but helps to detect potential bias, thereby supporting the interpretation of results.

##### Healthcare economic evaluation

A cost-minimization analysis with the nCP versus standard care will be conducted using health insurance billing data covering the period 1 week before and after the trauma, with a sensitivity analysis covering 1 month before and after the trauma. Alternatively, a cost-effectiveness analysis (CEA) can be conducted if the assumed non-inferiority of the nCP is not demonstrated in the clinical study. The comparison will be made using a Difference-in-Differences approach, with the cost-minimization analysis comparing only costs, and the CEA calculating the incremental cost-effectiveness ratio (ICER). Sensitivity analyses will be conducted to account for uncertainties in both approaches.

##### Process evaluation

To identify existing barriers for the implementation of the nCP and to develop strategies for overcoming these barriers, a mixed-methods approach combining qualitative and quantitative methods will be applied. Focus groups including parents, adolescents, physicians and ED nursing staff will be conducted using a semi-structured interview guideline for approximately 60 min per group. The recorded conversations will be qualitatively analyzed with MAXQDA software (VERBI Software GmbH, Berlin, Germany) and used to design standardized questionnaire for surveys with parents and ED staff. For the analysis of parent and staff surveys, uni- or bivariate descriptive analyses will be conducted. Depending on the type of variable, frequency distributions with central tendency and variability measures (mean, median, variance, standard deviation) will be compared. Statistical significance will be determined using appropriate statistical tests. Subgroup analyses and correlation analyses will also be performed. Healthcare policy recommendations to incorporate the nCP into statutory health insurance portfolios will be derived from the mixed methods approach and discussed in a stakeholder workshop.

### Monitoring

Patient recruitment will be monitored by the study coordinators monthly. In addition, site-specific standardized operating procedures will ensure implementation of the study protocol at each study site, monitored by the study coordination. In turn, the study coordination regularly reports to the funder about achieved recruitment and any study incidences quarterly.

A dedicated data monitoring committee is not installed, because the documentation by the local physicians includes mainly anamnestic and physical assessment data that can only be monitored by observing the physical examination itself. Due to the study population and the low target number of inclusions per month, logistics to ensure the monitoring of an adequate number of cases per study site is not possible to achieve within the scope of the funding. In turn, the eCRF is designed to be intuitive for physicians and includes predominantly single- or multiple-choice answers with cross validation of data items and validation ranges for continuous variables. Further, intensive training for study personnel will be provided by the study lead with ongoing in-person support throughout the entire study period. No interim analyses are planned but plausibility checks of entered data will be performed regularly. No stopping rules have been determined, because the care pathway provides the option of switching to standard care (hospitalization) and performing additional diagnostic and therapeutic measures at any time. Moreover, the co-primary outcomes are specifically designed to evaluate the success of reducing hospitalizations and its safety.

### Confidentiality

The study sites ensure pseudonymization and keep pseudonymization at their site, protected against unauthorized access. The study sites transmit personal data and the pseudonym of study participants who are insured with one of the participating health insurance companies to the respective health insurance company for the purpose of health economic evaluation. Besides this, only pseudonymized data are transferred with no possibility for the study lead or any non-local investigator to identify participants.

### Ancillary and post-trial care

No exams are conducted beyond those indicated according to clinical routine. For that reason, no findings will be generated that would otherwise not have been detected. After the end of the intervention in the ED, patients are automatically transferred back to standard care pathways. If any additional intervention is necessary during the monitoring period in the ED, this will also be performed according to standard care pathways. All complications detected during the nCP or after the end of the intervention will be treated within standard care pathways.

### Dissemination

The trial results will be published as demanded by the funding source within a structured project evaluation. Besides, all results will be published open access in scientific peer-reviewed journals. Authorship eligibility is based on ICMJE criteria and includes all active contributors of the project. Further details are specified in the project’s publication guideline that will be made publicly available after being agreed on by all parties. The participant level dataset will not be made publicly available, but secondary analyses can be requested by participating parties in conjunction with study investigators according to the publication guideline.

## Discussion

The SaVeBRAIN.Kids trial addresses a relevant healthcare challenge in Germany, where hospitalization rates for pediatric TBI drastically exceed rates in similar healthcare systems. The disparity likely reflects the use of non-standardized care pathways along with economic incentives posed by the diagnose related group reimbursement system. The study will be conducted using a cluster-randomized stepped-wedge design, allowing to integrate the novel care pathway into existing clinical settings at the same time as levelling out seasonal variations of TBI incidence.

Within the German healthcare system, the nCP represents a paradigm shift in mild TBI management for low-risk pediatric, emphasizing standardized assessment, structured observation in the emergency department, and technology-assisted home monitoring. This approach aligns with up-to-date international best practice while addressing country-specific decision-making processes that cause high hospitalization rates. By relying on evidence-based risk stratification and implementing standardized treatment/monitoring protocols, we aim to maintain medical safety while reducing unnecessary hospitalizations.

The dual co-primary outcome design balances effectiveness (hospitalization rate) with safety (unplanned re-visits), reflecting the need to achieve both cost-efficiency and non-inferiority with respect to clinical outcomes. The comprehensive economic evaluation will provide important information on cost-effectiveness that could inform broader implementation decisions for implementation into routine care. Including retrospective data analysis and process evaluation strengthens the study by providing context to interpret results and identify implementation barriers.

Limitations of the study include the lack of blinding during the intervention for both clinicians and parents, which is unavoidable given the study design. Additionally, the 72-h follow-up period may not capture all delayed complications, making an additional passive surveillance necessary. For the current study, the data entered into the parent app will not be viewable in real-time by the attending physicians to avoid the app falling under the medical device regulation of the EU. For future commercialization and implementation into routine care as a medical product, the integration of real-time data collection via the parent app may represent a particularly innovative approach to post-discharge surveillance.

If successful, the SaVeBRAIN.Kids trial has the potential to impact pediatric TBI management in Germany by reducing unnecessary hospitalizations while maintaining patient safety. The findings could provide a template to address other pediatric or adult conditions where standardized decision-making algorithms and technology-assisted monitoring benefit from optimizing resource utilization without compromising patient outcomes.

## Supplementary Information

Additional file 1: SPIRIT checklist.

Additional file 2: Supplementary figure: Schedule of enrolment, interventions, and assessments.

## Data Availability

After completion of the SaVeBRAIN.Kids study, a fully anonymized dataset will be prepared for secondary analyses and will be transferred from the TTP to the clinical parties of the study’s core team. Access to the final datasets will be granted for the corresponding evaluators and researchers belonging to the study management (Department of Pediatrics I at UK Essen and Department of Neuropediatrics at LMU). Secondary analyses can be requested as per publication guideline, after the main findings have been published.

## Abbreviations

CEA: Cost-effectiveness analysis
CPO: Co-primary outcome
CY: Child-years
eCRF: Electronic case report forms
ED: Emergency department
GLMM: Generalized linear mixed models
ICC: Intracluster correlation coefficient
ICER: Incremental cost-effectiveness ratio
MAR: Missing at random
MNAR: Missing not at random
nCP: Novel care pathway
SAP: Statistical analysis plan
sCP: Standard care pathway
TBI: Traumatic brain injury
TTP: Trusted third party
