# Supplementary material for: SaVeBRAIN.Kids—study protocol for a cluster-randomized stepped-wedge trial to reduce hospitalizations for mild traumatic brain injury in children in Germany
Source: Trials. 2025 Oct 30;26:454. doi: 10.1186/s13063-025-09240-8 (PMC12577025; doi:10.1186/s13063-025-09240-8)
Supplement: Supplementary file 2 — Additional file 2: Supplementary figure: Schedule of enrolment, interventions, and assessments. [file 13063_2025_9240_MOESM2_ESM.docx]

Supplementary figure: Schedule of enrolment, interventions, and assessments.

|  | **STUDY PERIOD** | | | | | |
| --- | --- | --- | --- | --- | --- | --- |
|  | **Allocation of center** | **Eligibility Screen** | **Post-allocation** | | | **Close-out** |
| **TIMEPOINT*** | ***-t_1_*** | **0** | ***t_1_*** | ***t_2_*** | ***t_3-7_*** | ***t_8_*** |
| **ENROLMENT:** |  |  |  |  |  |  |
| **Eligibility screen** |  | X |  |  |  |  |
| **Informed consent** |  | X |  |  |  |  |
| ***Decision on management (inpatient vs. outpatient)*** |  |  | X |  |  |  |
| **Allocation** | X |  |  |  |  |  |
| **INTERVENTIONS:** |  |  |  |  |  |  |
| ***Monitoring in the ED (optional, intervention group)*** |  |  |  |  |  |  |
| ***Discharge from ED*** |  |  |  | X |  |  |
| **ASSESSMENTS:** |  |  |  |  |  |  |
| ***History and physical exam*** |  | X |  |  |  |  |
| ***Safety assessment (parent-reported, only intervention group if no hospital admission)*** |  |  |  |  | X |  |
| ***Primary and secondary outcome assessment*** |  |  |  |  |  | X |

*t_1_: Directly after enrolment, t_2_: discharge from the emergency department (ED), t_3-7_: observation timepoints during home monitoring (2, 6, 12, 24, and 48 hours), t8: follow-up 72 hours after discharge from ED
